# Failure of ultrasound to diagnose a giant ovarian cyst: a case report

**DOI:** 10.4076/1757-1626-2-6909

**Published:** 2009-07-22

**Authors:** Themistoklis Mikos, George P Tabakoudis, George Pados, Nikos P Eugenidis, Efstratios Assimakopoulos

**Affiliations:** 1 1^st^ Department of Obstetrics & Gynecology, Aristotle University of Thessaloniki, Papageorgiou General HospitalPeriferiaki Odos Neas Efkarpias, 56 429, ThessalonikiGreece; 2 2^nd^ Propedeutic Department of Medicine, Aristotle University of Thessaloniki, Ippokrateion General HospitalThessalonikiGreece

## Abstract

Ultrasonography is the method of choice in the diagnosis of ovarian cysts. In this case report, a cyst of enormous volume (>35 litres) was limiting the application of ultrasound techniques giving the false impression of ascites. A 55-year-old woman was finally diagnosed as having a giant ovarian mucosal-serosal cystadenoma of borderline potential after undergoing a total abdominal hysterectomy with salpingo-oophorectomy and excision of the cyst. In the literature, similar conditions have been described with the term ‘empty abdomen’.

## Introduction

Ovarian cysts rarely grow immense. Ultrasound scan examination permits early detection and appropriate treatment. Occasionally, ovarian cysts reach enormous dimensions without raising any symptom. A few cases of giant ovarian cysts have been sporadically reported in the literature [1-4]. Differential diagnosis with ascites is a major concern during the management of these cysts.

A case of a giant ovarian cyst mimicking ascites in a 59-year-woman is presented. Emphasis is drawn on the initial failure of ultrasound to assist in the diagnosis due to the enormous size of the cyst that hampered the application of ultrasound techniques.

## Case presentation

**Figure 1.**
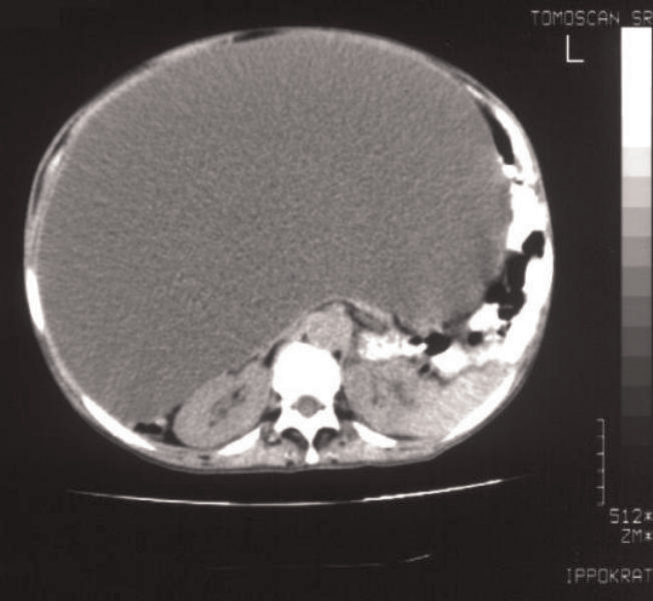
Initial computed tomogram of the abdomen of the patient with a giant ovarian cyst mimicking ascites.

**Figure 2.**
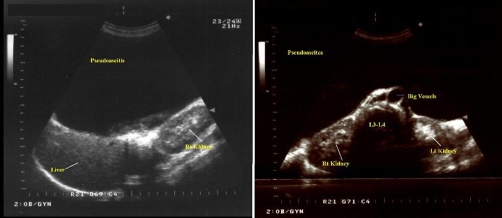
Transabdominal sonogram after paracentesis of the lesion. **(Left)** The liver and the right kidney are repelled to the posterior wall of the abdomen. The distance between the probe and the abdmominal organs is more than 13 cm. **(Right)** The large retroperitoneal vessels, the lumbar vertebrae and the two kidneys are seen.

**Figure 3.**
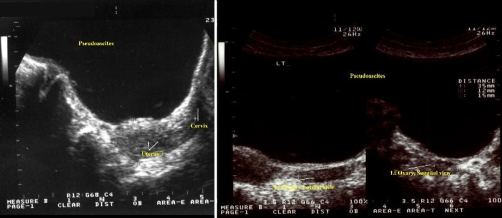
Transabdominal sonogram of **(Left)** the uterus and **(Right)** the left ovary of the patient.

**Figure 4.**
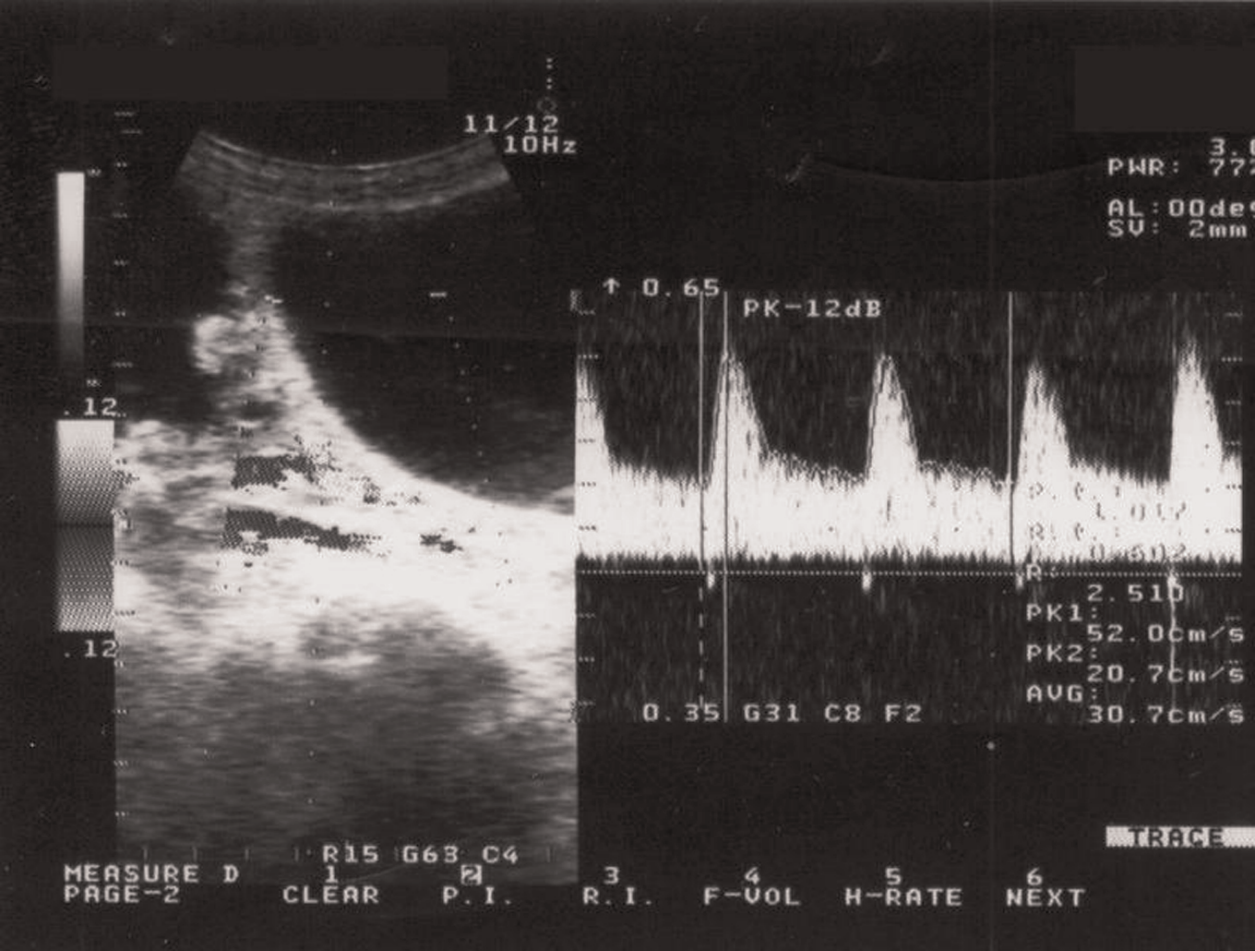
Transabdominal triplex sonogram of the vasculature of the right ovary after paracentesis of the giant cyst. The pulsatility index and the resistance index of the Doppler waveform of the vessels is 1.017 and 0.602 respectively.

A 59-year-old, Caucasian (Greek origin), multiparous (Gravida 3, Para2, two spontaneous vaginal deliveries, housewife) was hospitalised in the medical ward complaining of dyspnoea and abdominal distension progressing during the last eight months. She was 1.62 m high, and she was weighing 73 kg at admission. She had no significant medical and surgical history, and she did not smoke or drink. Initial abdominal imaging with computed tomography and abdominal ultrasound showed huge ascites and suggested no signs of ovarian or other intra-abdominal pathology (Figure 1). Biochemistry was normal except for liver function tests that appeared moderately elevated. Tumor markers were normal except CA-125 which was 300 U/ml. Serum-ascites albumin gradient was 1.30. There was a history of hepatitis B infection, but at that time the patient was receiving no treatment for the liver. The patient underwent serial drainage of approximately 35 litres of fluid to relieve symptoms. Cytology of the aspirated fluid was normal. The patient was discharged on spironolactone and furosemide and was examined on an outpatient basis. Six months later she was admitted again for recurrence of symptoms. Further drainage removed another 14 litres. Repeat CA-125 was within normal limits. A new sonographic approach (ALOKA, SSD-1700, transabdominal transducer 3.5-5.5 MHz) after paracentesis revealed normal uterus and left ovary, an increased vascularisation of the right adnexa on the colour flow mode, and the liver characteristically repelled on the back wall of peritoneal cavity, findings compatible with those of a giant ovarian cyst (Figures 2-4). A new computed tomography confirmed the sonographic diagnosis. At subsequent laparotomy the patient underwent excision of the giant cyst and a total abdominal hysterectomy with bilateral salpingo-oophorectomy. Pathology examination showed that the lesion was a mucosal-serosal ovarian cystadenoma of borderline malignant potential. The patient had an uneventful postoperative course and after 3 years of follow up she is doing well.

## Discussion

Giant ovarian cysts mimicking ascites have been rarely reported in the literature. Similar conditions may arise from omentum, mesenterium, or retroperitoneal structures, and the differential diagnosis includes ascites, urinary retention, bladder diverticulum, hydronephrosis, pancreatic pseudocysts, and large uterine tumors [4-10].

It is obvious from the history of the patient that initial sonographic approach failed to diagnose correctly the cyst. However, specific sonographic features that are pathognomonic of giant ovarian cysts were met after paracentesis of the cyst. First, the sonographic appearance of the intraperitoneal organs was highly suggestive of a giant growth. The liver was compressed just above the right kidney (and not free floating), a finding that contradicted to the diagnosis of free intraperitoneal fluid. The absence of other intraperitoneal structures, such as floating bowel loops within the abdomen, was another sign of a cystic mass repelling any other abdominal structure away. Second, the use of colour flow was highly suggestive of a neoplastic disorder appearing from the ovary. The colour flow is a sonographic feature that detects movement and is used in the vascular imaging and in producing Doppler waveforms. In the presented case, the colour flow detected a highly vascularised area within the lateral surface of the peritoneal wall. No normal intraoperative organs produce such an intense signal, so the presence of vasculature supplying a huge cyst, probably of ovarian origin, was further suspected. The combination of ultrasound techniques (morphologic assessment, color Doppler flow imaging, and Doppler indexes) have been found to perform well (sensitivity = 84%, specificity = 82%, positive likelihood ratio = 4.69) compared to computed tomography (sensitivity = 81%, specificity = 87%, positive likelihood ratio = 6.81) in the diagnosis of ovarian lesions [11]. Computed tomography had similar findings to the ultrasound suggesting of an ovarian mass repelling the rest of the intraperitoneal organs. Clinical computed tomography images indicate that high accumulation of intraperitoneal fluid can increased the pressure and actually displaces bowel loops to one side of the abdomen and gives a compressed appearance (tense ascites). This is an atypical presentation of ascites that can be easily confused with a mass [12].

The sonographic features described above are pathognomonic of a giant ovarian cyst. The term ‘empty abdomen’ has been used to describe the sonographic appearance of similar conditions.

## Conclusions

In conclusion, giant ovarian cysts should always be considered in the differential diagnosis of conditions such as ascites. Two dimensional ultrasound appears to be a cost-effective and accurate technique. The application of extra features such as the colour flow and the Doppler waveforms enhance its diagnostic accuracy. There are cases where drainage of such cysts helps in the application of the sonographic techniques, otherwise the volume of the cysts is a limit to the diagnostic sonographic approach.
